# The Effect of Wearing an N95 Face Mask on Intraocular Pressure

**DOI:** 10.18502/jovr.v20.16584

**Published:** 2025-12-08

**Authors:** Naveed Nilforushan, Farhad Najafzadeh, Samira Chai Bakhsh, Masoume Sharfinejad

**Affiliations:** ^1^Department of Ophthalmology, Eye Research Center, Rassoul Akram Hospital, Iran University of Medical Sciences, Tehran, Iran; ^2^Department of Ophthalmology, Nikookari Eye Hospital, Tabriz University of Medical Sciences, Tabriz, Iran; ^3^Rajaie Cardiovascular Medical and Research Center, Iran University of Medical Sciences, Tehran, Iran

**Keywords:** IOP, N95 Face Mask, Oxygen Saturation

## Abstract

**Purpose:**

To evaluate the effect of wearing an N95 mask on intraocular pressure.

**Methods:**

This cross-sectional study enrolled 34 medical staff volunteers. After baseline eye examinations, all participants were asked to wear an N95 mask for 1 hour. Then, O ${}_{2}$ saturation, pulse rate, and blood pressure were evaluated, and sequential IOP measurements were performed using both iCare and Goldmann applanation tonometers. All measurements were taken again 1 hour after the mask was removed. Finally, all information was collected for statistical analysis.

**Results:**

A total of 34 participants (68 eyes) with a mean ± SD age of 38.97 (12.41) years were included. The mean value of IOP/GAT increased significantly by 1.20 mmHg after wearing the mask (12.50 to 13.70 mmHg, *P*-value < 0.001) and then decreased significantly by 1.27 mmHg after removing the mask (13.70 to 12.43 mmHg, *P*-value < 0.001). The mean O ${}_{2}$ saturation increased significantly from 96.4 with the mask to 97.05 after mask removal (*P*-value < 0.001). Although the mean pulse rate decreased by about 1.5 units after removing the mask, the difference was not significant. The mean blood pressure decreased after removing the mask; however, the change was not statistically significant. Additionally, no significant relationship was found between the change in IOP and O ${}_{2}$ saturation.

**Conclusion:**

The use of N95 face masks could substantially increase IOP and reduce O ${}_{2}$ saturation.

##  INTRODUCTION

The SARS-CoV-2 pandemic dramatically changed our daily lives. Causing acute respiratory syndrome, this virus resulted in the hospitalization and death of a large number of people worldwide.^[1,2]^ It is a highly contagious disease due to its small size, ranging from 60 to 140 nm,^[3]^ and its various transmission methods, including the inhalation of airborne particles and droplets.^[4]^ Affected patients cough and sneeze in hospital environments, increasing the risk of infection in medical workers by 12 times.^[5]^ There are various types of respiratory masks suitable for medical settings, including surgical masks, N95 masks, P100 masks, and multi-layered cotton masks.^[12,13,14,15,16]^ The high capacity of the N95 mask to filter small particles has made it the most commonly used mask in hospitals. The letter N in N95 represents "no resistance to oil," and the number 95 also shows that this type of mask, in the worst case, prevents the passage of 95% of particles > 0.3 microns.^[6]^

Preventive measures to reduce the transmission of COVID-19 include using a face mask, social distancing, and vaccination.^[6]^ The use of personal protective equipment is especially important for healthcare workers who are directly exposed to this virus. According to the US Centers for Disease Control and Prevention, proper use of N95 masks is the most important measure healthcare workers can take to prevent the spread of COVID-19.^[7]^ However, some research has shown that prolonged use of N95 masks can cause hypoxia and retention of carbon dioxide (CO ${}_{2}$ ).^[8]^ Other studies have stated that hypoxia and hypercapnia can directly increase intraocular pressure (IOP) and, by causing hyperemia and thickening of the choroidal plexus, ultimately lead to changes in IOP.^[9,10,11]^

Although wearing a mask can affect the saturation of oxygen (O ${}_{2}$ ) and CO ${}_{2}$ and, thereby, change the IOP, to the best of our knowledge, no specific study has explored the effects of N95 masks on eye pressure.

Considering the common use of these masks by medical personnel in hospital settings, the present study aimed to investigate the effect of using N95 masks on eye pressure.

##  METHODS

This cross-sectional study enrolled 34 volunteer employees from Rassoul Akram Hospital in Tehran, Iran. The study was conducted in accordance with the principles of the Declaration of Helsinki and has been registered with the Ethics Committee of the Iran University of Medical Sciences (IR.IUMS.FMD.REC.1401.222). First, the objectives and methods of the study were explained to all participants, and their written informed consent was obtained thereafter. Additionally, to be eligible for the study, all participants must have received two doses of the COVID-19 vaccine, with the final dose administered within the last 4 months. The exclusion criteria were age under 18 years, high refractive error (astigmatism > 3 diopters; myopia, hyperopia > 5 diopters), history of any type of eye surgery, history of glaucoma, presence of any kind of corneal scar, inability to wear a mask, and acute or uncontrolled cardiac and respiratory diseases. Participants' demographic characteristics, including age, sex, height, weight, and records of systemic diseases, were gathered.

Next, all participants received complete ophthalmic examinations, including refraction, best-corrected visual acuity, slit lamp examination, fundus examination, IOP assessment, and corneal thickness measurement with the Pentacam device. To measure the IOP, participants were first seated and then asked to look directly at a fixed target at a specified distance. The IOP was first measured with the iCare 200 device (iCare USA Inc., Raleigh, NC). At least two measurements were performed in each eye. If the difference between the two measurements was > 2 mmHg, a third measurement was taken, and the average pressure for each eye was recorded as the final pressure. Then, within 5 minutes, IOP was measured again using the Goldmann applanation tonometry (GAT) device as follows: a tetracaine drop was used for local anesthesia, and then a fluorescein paper strip was used to stain the tears. Participants were placed in a suitable position behind the slit lamp, and IOP was measured.

During the IOP measurement, a 30-second interval was allowed between measurements of the two eyes. In this way, the pressure of the right eye was measured first, followed by a 30-second rest period, and then the pressure of the left eye was measured. Measurements were performed at least twice in each eye. If a difference of > 2 mmHg was observed, measurements were repeated a third time, and the average pressure for each eye was recorded as the final pressure. All examinations were done between 9:00 AM and 11:00 AM.

Then, participants were given the same brand of N95 masks (KN95 Three-Dimensional Protective Face Mask, Guangdong Zhizhen Biological Medicine CO., Ltd, China) and asked to wear them for 1 hour in the clinic environment. Afterward, O ${}_{2}$ saturation and pulse rate were measured using a pulse oximetry device, and blood pressure was measured with a standard mercury sphygmomanometer (model 7670, Welch Allyn, a brand of Baxter International Inc., Skaneateles Falls, NY, USA). IOP was measured with the iCare and GAT devices using the previously mentioned method. While measuring eye pressure with the GAT, one of the colleagues was careful to ensure that the tonometer did not touch the patient's mask to avoid any measurement errors.

After all these steps, the mask was removed, and the participants were asked to stay in an isolated area of the clinic for 1 hour. The IOP was measured again using the iCare and GAT devices with the same method as before. O ${}_{2}$ saturation and pulse rate measurements were taken using a pulse oximetry device.

Finally, all the information was collected, and statistical analyses were done by SPSS software version 22. A two-sided Student *t*-test was used to compare two groups for continuous variables, and a two-sample test and Fisher's exact test were used for discrete variables. A *P*-value < 0.05 was considered significant. The number of samples, consisting of 34 people, was determined by considering a statistical power of 90% and a significance level of 0.05.

##  RESULTS

A total of 34 participants (68 eyes) with a mean ± SD age of 38.97 ± 12.41 years were included in the study. Table 1 shows the demographic and clinical features of the participants and their eyes. Fifteen (44%) participants were male. The mean ± SD body mass index (BMI) was 26.72 ± 4.46. The clinical characteristics are described in total and by right and left eyes. The mean ± SD baseline IOP measured by GAT was 12.5 ± 1.57 and 14.24 ± 1.59 mmHg by iCare.

**Figure 1:**
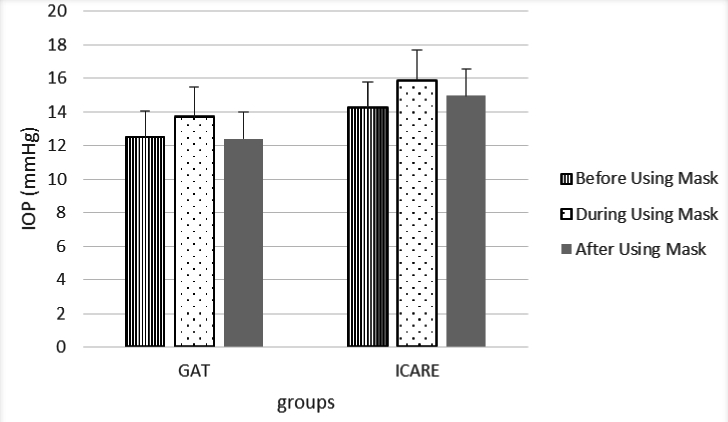
IOP values at baseline, while using an N95 mask, and after removing it.

The mean and standard deviations of the outcomes during the experiment are presented in Table 2. In total, the mean value of IOP/GAT increased significantly by 1.20 mmHg after wearing the mask (12.50 to 13.70 mmHg, *P* < 0.001) and then decreased significantly by 1.27 mmHg after removing the mask (13.70 to 12.43 mmHg, *P* < 0.001). Additionally, the mean value of IOP/iCare increased significantly by 1.64 mmHg after wearing the mask (14.24 to 15.88 mmHg, *P* < 0.001) and then decreased significantly by 0.92 mmHg after removing the mask (15.88 to 14.96 mmHg, *P* < 0.001). Figure 1 illustrates IOP values at baseline, during the use of an N95 mask, and after removing it.

The mean value of O ${}_{2}$ increased significantly from 96.4 ± 1.46 with the mask to 97.05 ± 1.2 after removing the mask (*P* < 0.001). Although the mean pulse rate decreased by about 1.5 units after removing the mask, no significant difference was detected (76.74 ± 9.95 to 78.18 ± 10.68, *P *= 0.391). Additionally, the mean blood pressure decreased after removing the mask, but the change was not significant (86.01 ± 13.75 to 84.28 ± 13.22, *P *= 0.077).

The relationship between IOP and O2 changes (before and after using the mask) was examined. The results showed no statistically significant relationship between IOP and O ${}_{2}$ changes, as assessed using iCare, and no significant association was observed using GAT (*P *= 0.770 for iCare and *P* = 0.745 for GAT).

The variables were repeatedly adjusted to consider the effects of clinical and demographic factors on the outcome. The results showed no remarkable change in the analyses results.

##  DISCUSSION

Wearing a face mask was one of the most effective ways to prevent COVID-19 during the pandemic, and even after receiving several vaccine doses, it remained one of the most effective methods to prevent it.^[12,13,14]^ Barasheed et al systematically analyzed the use and effectiveness of masks by integrating 12,710 samples from more than 50 countries worldwide and found that the use of masks in crowded places can reduce the risk of respiratory infections.^[14]^ However, while the effectiveness of masks has been proven, the effects of wearing masks remain unclear. For example, Sammito et al showed that the level of changes in blood oxygen before and after wearing FFP masks is insignificant,^[22]^ but Lee et al found that wearing an FFP2/N95 mask leads to a 13% decrease in VO2max and a 37% reduction in air exchange volume.^[23]^

**Table 1:** The clinical and demographic characteristics of participants (*n* = 34)

**Variable**	**Mean ± SD**
Gender	
Male (*n*%)	15 (44%)
Female (*n*%)	19 (56%)
Age(year)	38.97 ± 12.41
BMI(kg/m ${}^{2}$ )	26.72 ± 4.46
CCT (µm)	
OD	527.88 ± 31.44
OS	529.44 ± 32.21
Total	528.66 ± 31.48
Refraction (sphere)	
OD	–1.23 ± 2.34
OS	–1.30 ± 2.09
Total	–1.26 ± 2.19
UCVA (LogMAR)	
OD	0.34 ± 0.37
OS	0.32 ± 0.39
Total	0.33 ± 0.38
BCVA (LogMAR)	
OD	0.01 ± 0.05
OS	0.03 ± 0.15
Total	0.02 ± 0.11
VCDR (decimal)	
OD	0.24 ± 0.17
OS	0.22 ± 0.14
Total	0.23 ± 0.15
IOP/GAT (mmHg) baseline	
OD	12.5 ± 1.56
OS	12.5 ± 1.58
Total	12.5 ± 1.57
IOP/ICARE (mmHg) baseline	
OD	14.21 ± 1.40
OS	14.26 ± 1.77
Total	14.24 ± 1.59
BMI, body mass index; CCT, central corneal thickness; OD, oculus dexter (right eye); OS, oculus sinister (left eye); UCVA, uncorrected visual acuity; LogMAR, logarithm of the minimum angle of resolution; BCVA, best-corrected visual acuity; VCDR, vertical cup-to-disc ratio; IOP, intraocular pressure; GAT, Goldmann applanation tonometer; ICARE, iCare tonometer – a portable device that uses rebound tonometry; mmHg, millimeters of mercury.	

**Table 2:** IOP changes before, during, and after using an N95 mask

**IOP**	**Before mask**	**During mask use**	**After mask use**	***P*** **-value** * *	***P*** **-value** * *
	**Mean ( SD)**	**Mean ( SD)**	**Mean ( SD)**	**(baseline vs. during mask use)**	**(during vs. after mask use)**
IOP/GAT (mmHg)					
OD	12.51 (1.56)	13.64 (1.83)	12.41 (1.58)	< 0.001*	< 0.001*
OS	12.59 (1.58)	13.77 (1.77)	12.44 (1.58)	< 0.001*	< 0.001*
Total	12.50 (1.57)	13.70 (1.79)	12.43 (1.57)	< 0.001*	< 0.001*
IOP/ICARE (mmHg)					
OD	14.21 (1.40)	15.79 (1.92)	14.85 (1.73)	< 0.001*	< 0.001*
OS	14.26 (1.77)	15.96 (2.04)	15.06 (1.71)	< 0.001*	< 0.001*
Total	14.24 (1.59)	15.88 (1.97)	14.96 (1.71)	< 0.001*	< 0.001*
ICARE, iCare tonometer – a portable device that uses rebound tonometry; mmHg, millimeters of mercury; IOP, intraocular pressure; SD. standard deviation; GAT, Goldmann applanation tonometer.					

Surgical masks are the most commonly used type of personal protective equipment. Although surgical masks are typically loose-fitting, and N95 respirators are disposable and vary in quality and level of protection, they both create a physical barrier between the wearer and potential contaminants in the environment. N95 masks have the advantage of blocking at least 95% of both small aerosols ( < 5 μ m) and larger droplets (5–50 μ m).^[24]^

In this study, we examined IOP changes along with changes in O ${}_{2}$ saturation with and without a mask in order to explore the potential effect of mask use on IOP. The results showed that mask use significantly increased IOP and slightly reduced O ${}_{2}$ saturation. However, a direct relationship between changes in IOP and blood oxygen could not be proven. The reason was that many other factors, such as blood carbon dioxide (CO ${}_{2}$ ) levels or hormonal factors, that could have been effective in these IOP changes were not investigated due to the invasiveness of their evaluation methods.

Currently, studies on how wearing a mask affects IOP and its possible mechanisms are limited, with conflicting results, and more comprehensive investigations are needed.

In a study by Najmanova et al, IOP increased significantly in response to short-term normobaric hypoxia and returned to baseline 7 minutes after hypoxia in well-controlled environmental conditions. They found that IOP increased by 1.2 ± 1.2 mmHg and 2.3 ± 0.9 mmHg, respectively, at 4 and 10 minutes during the hypoxia period, and almost returned to the initial state after 7 minutes. They concluded that hypoxia-induced changes in IOP were significantly associated with changes in arterial oxygen saturation, and a strong inverse relationship existed between oxygen saturation level and IOP. In contrast, the relationship between baseline IOP and initial heart rate was insignificant.^[19]^ In our study, we did not observe this inverse relationship between oxygen changes and IOP.

Janicijevic et al investigated the use of masks and their effect on IOP after activity. The results showed that using face masks and engaging in physical activities (such as work or sports) can increase IOP by 1 to 2 mmHg, and this increase can have a compounding effect on individuals prone to elevated IOP, such as patients with glaucoma.^[15]^

Vera J et al reported that FFP2 masks increased the IOP response during dynamic and isometric biceps curl exercises. They proposed that, if possible, patients with glaucoma should limit the use of FFP2 masks during resistance exercise. Therefore, they concluded that using this mask could pose risks and cause more damage to personnel in medical systems due to its continuous use with physical activities, especially in people with diseases such as glaucoma.^[21]^

In another study, Janicijevic et al investigated the effect of using surgical masks and FFP2/N95 on IOP during a 400-meter walking protocol among patients with primary open-angle glaucoma. Using an iCare tonometer, the authors reported that the use of different masks did not affect the level of IOP at rest (measurements at baseline and recovery). However, they found that wearing an FFP2/N95 mask caused a small but statistically significant change (mean difference of 1–2 mmHg) during physical activity, and IOP increased significantly when this mask was used compared to the surgical mask and the control condition.^[20]^ In contrast, our study showed that wearing a mask without engaging in physical activity can also affect IOP; one reason for this observation could be that all participants used N95 masks in our study.

Another mechanism that can contribute to IOP differences is the change in CO ${}_{2}$ saturation. Several studies have shown a direct relationship between an increase in CO ${}_{2}$ and an increase in IOP.^[16]^ Additionally, other studies have demonstrated that the use of face masks in combination with physical activities can increase CO ${}_{2}$ , leading to a rise in IOP.^[17,18]^

New cases of COVID-19 continue to be reported, and widespread use of masks is still recommended. On the other hand, only limited studies with conflicting results have been conducted on the impact of using face masks and IOP changes. In this context, the present study showed that the use of N95 masks increases IOP, which is accompanied by a decrease in O ${}_{2}$ saturation.

Despite the increasing COVID-19 vaccination rates and a decrease in the prevalence and occurrence of the disease, as indicated by epidemiological data, wearing a mask—especially for medical personnel—remains the most effective way to prevent the disease. This is because of the continuous evolution of the virus and the lack of vaccine protection against new strains. Nevertheless, attention must be given to the potential side effects of prolonged mask use. The current study noted an increase in IOP when the mask was used for 1 hour, but the effect of more prolonged use, especially in people with a history of glaucoma or at high risk for glaucoma, remains unclear.

In the present study, an attempt was made to investigate the short-term effect of N95 mask use on eye pressure and other factors such as blood oxygen, pulse rate, and blood pressure. However, this study had several limitations.

• We included only healthy young people as participants, which limits the generalizability of the results to other individuals or patients with glaucoma.

• We did not examine CO ${}_{2}$ changes, which can be a significant factor influencing IOP changes. Checking CO ${}_{2}$ generally requires an arterial blood gas (ABG) test, which was not performed due to the aggressive nature of the procedure.

• We only assessed the effect of wearing the mask for 1 hour and did not explore its long-term use.

• Participants were asked to avoid vigorous physical activity during the evaluation; therefore, it is unclear how physical activities might affect the results.

Further studies addressing the aforementioned limitations would likely enhance our understanding of the impact of wearing N95 masks on IOP.

In summary, the data obtained from the present study showed that the use of N95 face masks by hospital medical personnel could cause a marked increase in IOP.

##  Financial Support and Sponsorship

None.

##  Conflicts of Interest

None.
